# Time Course of the Phenotype of Blood and Bone Marrow Monocytes and Macrophages in the Lung after Cigarette Smoke Exposure In Vivo

**DOI:** 10.3390/ijms18091940

**Published:** 2017-09-09

**Authors:** Camila Oliveira da Silva, Andréa Monte-Alto-Costa, Mariana Renovato-Martins, Filipe Jorge Viana Nascimento, Samuel dos Santos Valença, Vincent Lagente, Luís Cristóvão Pôrto, Tatiana Victoni

**Affiliations:** 1 Laboratório e Histocompatibilidade e Criopreservação, HLA/Universidade do Estado do Rio de Janeiro, Rio de Janeiro, RJ 20950-000, Brazil; camilavs162@hotmail.com (C.O.d.S.); filipej.bio@gmail.com (F.J.V.N.); lcporto@uerj.br (L.C.P.); 2 Laboratório de Reparo Tecidual, DHE/IBRAG/Universidade do Estado do Rio de Janeiro, Rio de Janeiro, RJ 20950-003, Brazil; andreamacosta@gmail.com; 3 Instituto de Biologia Roberto Alcântara Gomes, Universidade do Estado do Rio de Janeiro, Rio de Janeiro, RJ 20551-030, Brazil; m_renovatomartins@yahoo.com.br; 4 Laboratório de Biologia Redox, ICB/Universidade Federal do Rio de Janeiro, Rio de Janeiro, RJ 21941-902, Brazil; samuelv@hotmail.com; 5 Nutrition, Métabolismes et Cancer, NUMECAN Unité, Institut national de la santé et de la recherche médicale, INSERM 1241/Institut national de la recherche agronomique, INRA 1341/Université de Rennes 1, 35000 Rennes, France; vincent.lagente@univ-rennes1.fr

**Keywords:** COPD, macrophages, cigarette smoke, monocytes

## Abstract

Alveolar macrophages play a central role in the pathogenesis of chronic obstructive pulmonary disease (COPD). Monocytes are recruited from blood during inflammation and then mature into alveolar macrophages. The aim of this study was to investigate the effect of cigarette smoke (CS) at different times in lung macrophages and monocytes from blood and bone marrow in mice. Male mice (C57BL/6, *n* = 45) were divided into groups: control, CS 5 days, CS 14 days and CS 30 days. Five days’ CS exposure induced a pronounced influx of neutrophils and macrophages in the lung associated with increased levels of keratinocyte chemoattractant (KC), tumor necrosis factor-α (TNF-α), nitric oxide (NO) and matrix metalloproteinase (MMP)-12. After 14 days of CS exposure, neutrophil recruitment and cytokine production were greatly reduced. Moreover, chronic CS exposure led to increased recruitment of macrophages (with high expression of CD206), transforming growth factor-β (TGF-β) production as well as no detection of TNF-α, interleukin (IL)-6 and KC. CS can also change the monocyte phenotype in the blood and bone marrow, with an increase in Ly6C^low^ cells. These results show for the first time that CS can change not only macrophage polarization but also monocyte. These results suggest that continued recruitment of Ly6C^low^ monocytes may help the distinct renewing macrophage M2 population required for COPD progression.

## 1. Introduction

Chronic obstructive pulmonary disease (COPD) is a complex disease characterized by an inflammatory response to inhaled noxious particle gases [1]. The major risk factor for COPD, as has been proven in many studies, is exposure to cigarette smoke (CS). It is imputed as the initial trigger for the activation of cells of the innate immune system, such as epithelial cells and macrophages [2]. Various studies have shown an increase in the number of macrophages in the lungs of patients with COPD; moreover, the number of macrophages is associated with the progression of this disease [3,4]. Alveolar macrophages (AM) play a central role in the pathogenesis of COPD in both the initiation and resolution of the inflammatory response that can alter the normal lung structure [5]. AM can change their surface phenotypes and functional properties in response to exogenous stimuli and pathological processes [6]. Macrophages have been characterized as classically activated (M1) or alternatively activated (M2) based on surface receptors, gene signatures and secretion of inflammatory mediators. Gordon [6] described that lipopolysaccharides, interleukin-1β (IL-1β) and cytokines secreted by Th1 lymphocytes, such as interferon-γ (IFN-γ), induce phenotype M1, whereas Th2 lymphocyte cytokines, such as IL-4 and IL-13, promote the phenotype M2. Activated M1 macrophages produce proinflammatory cytokines such as tumor necrosis factor-α (TNF-α), IL-6 and IL-12, whereas M2 macrophages produce anti-inflammatory molecules such as IL-10 and transforming growth factor-β (TGF-β) [7,8,9,10].

The lung macrophages from COPD patients have been suggested to exhibit a M1 phenotype due to the high amount of M1 cytokines, such as TNF-α and IL-8, found in their bronchoalveolar lavage (BAL) [11]. Moreover, TNF-α has also been strongly involved in emphysema induced by cigarette smoke in mice [12,13]. Controversially, current clinical evidence has shown that cigarette smoke induces macrophage reprogramming toward M2 polarization in patients with COPD [14]. Increased expression of M2 markers (CD163, CD204 and CD206) has been shown in COPD patients [15,16,17], and up-regulated M2 cytokines, such as IL-10, TGF-β1 and TGF-β2, were observed in mouse macrophages in vitro after exposure to different concentrations of CS extract [18].

AM are present in the alveolar and interstitial septa; they are recruited from circulating monocytes during an inflammatory reaction and then mature into alveolar macrophages [19,20]. The existence of monocyte subsets with distinct roles in homeostasis and inflammation suggests specialized functions. Classical CD14^++^/CD16^−^ monocytes were better at proinflammatory cytokine secretion [21], phagocytosis and reactive oxygen species (ROS) production [22], and the non-classical CD14^+^/CD16^+^ cells resembled resident tissue macrophages with higher expression of MHC class II molecules at their surface [23]. In addition, Geissmann et al. [24] indicated that Ly6C^+^ mouse monocytes correspond to CD14^++^/CD16^−^ (classic) human monocytes, and Ly6C^−^ mouse monocytes correspond to CD14^+^/CD16^+^ (non-classical) human monocytes. The phenotype of these cells resembled monocytes found in the bone marrow [25]. Circulating monocytes exhibit phenotype heterogeneity, which will probably result in distinct macrophage phenotype differentiation [26]. Nevertheless, few studies have elucidated the role of CS in monocyte and macrophage polarization towards the progression or aggravation of COPD in vivo model. The aim of this study was to investigate the effects of CS at different times in lung macrophages and monocytes of blood and bone marrow in mice.

## 2. Results

### 2.1. Cigarette Smoke (CS) Exposure Induces Time-Dependent Cellular Inflammation and Destruction in the Airways

**Figure 1:**
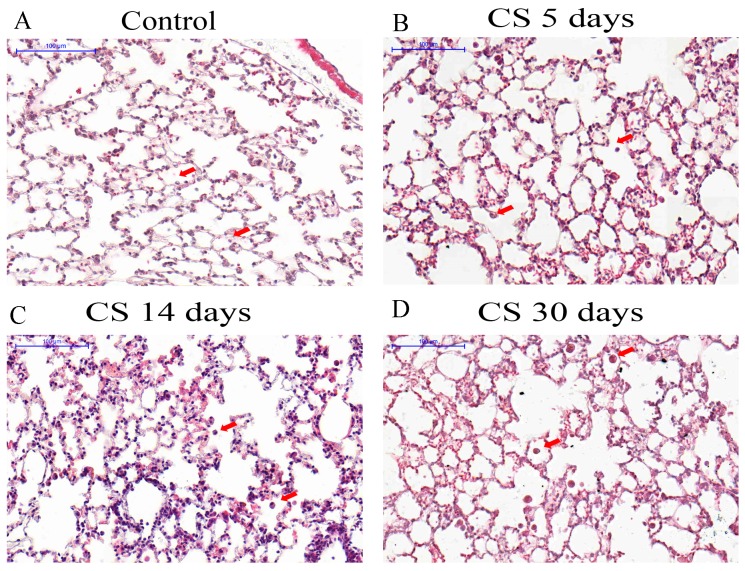
Photomicrographs of lung sections stained with hematoxylin and eosin. Bars are equal to 100 μm. (**A**) Control group (air exposed); (**B**) Mice exposed to cigarette smoke (CS) for 5 days; (**C**) Mice exposed to CS for 14 days; (**D**) Mice exposed to CS for 30 days. Arrowheads represent leukocytes in alveoli.

**Figure 2:**
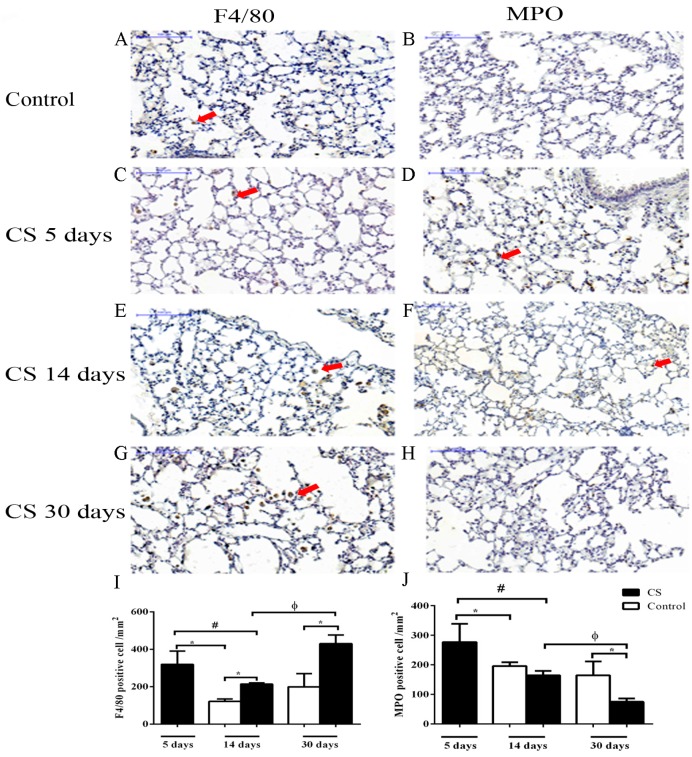
Effects of CS in cell populations in lung tissue. Mice were exposed to ambient air 14 and 30 days (**A** and **B**) or CS for 5 days (**C** and **D**), 14 days (**E** and **F**) and 30 days (**G** and **H**). Myeloperoxidase (MPO) expression was used as a neutrophil marker and F4/80 was used as a macrophage marker. Arrowheads represent positive cells. Bars are equal to 100 μm. * *p* < 0.05 when compared to control; ^#^ *p* < 0.05 compared to CS 5 days group; ^φ^ *p* < 0.05 compared to CS 14 days group. The values for all the measurements are expressed as the means ± SEM for 7–11 mice per group. All statistical analyses were performed with a one-way analysis of variance (ANOVA), followed by a Tukey’s *post hoc* test.

Lung sections were examined by light microscopy to observe the temporal effects of CS in lung tissue. Occasional alveolar macrophages were observed in control mice lung sections (Figure 1A). The lungs from CS 5 days presented cells in the alveoli and alveolar septa (Figure 1B) and cell infiltration was more pronounced in the lungs from mice exposed to CS for 14 days (Figure 1C) and CS 30 days (Figure 1D) when inflammatory cells were more numerous. To investigate the type of inflammatory cell found in the lung tissue after CS exposure, the number of F4/80-positive macrophages (Figure 2A,C,E,G,I) and myeloperoxidase (MPO)-positive neutrophils (Figure 2B,D,F,H,J) were quantified. We observed an increase in macrophages, with a peak of the cell number at 30 days of CS exposure (Figure 2I). The neutrophil number was only increased compared to control group after 5 days CS exposure (Figure 2J). In contrast, the number of these cells was lower in the 30 days of CS group than in the control group.

### 2.2. Effects of CS on Cytokine, NO Release and MMP-12 Expression in Lung Tissue

**Figure 3:**
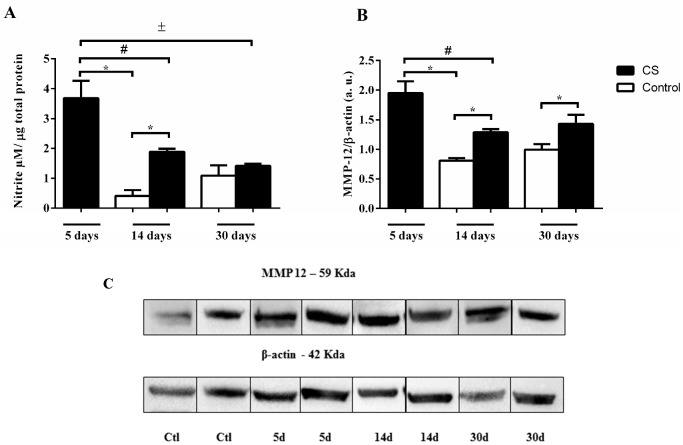
Effects of CS on nitrite levels and matrix metalloproteinase-12 (MMP-12) expression in the lung. The mice were exposed to 5, 14 and 30 days of CS or 14 and 30 days of ambient air and their lungs were harvested for detection of (**A**) the nitrite levels and (**B**) MMP-12 levels, (**C**) MMP-12 expression. All statistical analyses were performed with a one-way analysis of variance (ANOVA), followed by a Tukey’s *post hoc* test. The data represent the means ± SEM of 4–10 mice per group. * *p* < 0.05 compared to control; ^#^ *p* < 0.05 compared to 5 days group; ^±^ *p* < 0.05 compared to 30 days group.

Nitric oxide (NO) production is usually used to denote M1 macrophages, a certain quantity of NO to perform their functions, such as destroying invading pathogens, killing tumor cells and removing foreign materials. Conversely, M2 macrophages produce lower NO levels. Therefore, we evaluated if CS exposure could affect the synthesis of NO at different times. The nitrite levels were increased 5 days after CS but reduced 14 days later; after 30 days, no difference was observed compared to control (Figure 3A). In addition, the protein levels of matrix metalloproteinase-12 (MMP-12) leads to tissue degradation that has been suggested as M2 macrophages. MMP-12 was increased in the CS 5, 14 and 30 days of exposure when compared to the control (Figure 3B,C).

**Figure 4:**
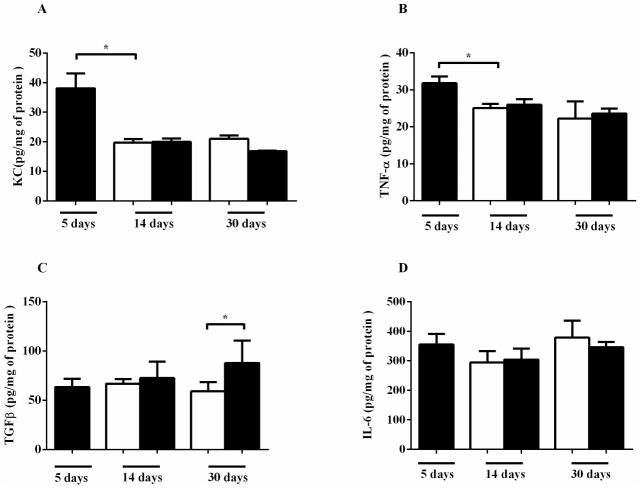
Effects of CS on cytokine production in the lung. The mice were exposed to 5, 14 and 30 days of CS (black bars) or ambient air (white bars) for 14 and 30 days and lung homogenates were isolated. The cytokines were measured by ELISA for (**A**) keratinocyte chemoattractant (KC), (**B**) interleukin-6 (IL-6), (**C**) tumor necrosis factor-α (TNF-α) and (**D**) transforming growth factor-β (TGF-β). Data represent the means ± SEM of 7–10 mice per group. * *p* < 0.05 compared to control. All statistical analyses were performed with a one-way ANOVA, followed by a Tukey’s *post hoc* test.

The inflammatory cell recruitment in COPD has been associated with increased levels of KC (keratinocyte chemoattractant), TNF-α and IL-6 in the lung. Therefore, we measured the levels of these cytokines after different CS exposure times. CS exposure for 5 days induced KC production in lung homogenate (Figure 4A). The production of TNF-α was also increased in the CS 5 day group (Figure 4B). In contrast, no marked difference in IL-6 after CS exposure (Figure 4C) and TGF-β was detected only after 30 days of CS exposure (Figure 4D).

### 2.3. Effects of CS Macrophage Phenotypes in Bronchoalveolar Lavage (BAL) after Cigarette Smoke Exposure

**Figure 5:**
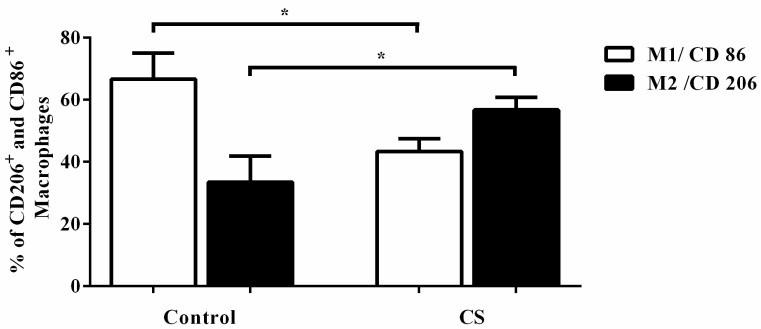
Effects of CS on the expression of CD86 (M1 marker) and CD206 (M2 marker) in bronchoalveolar lavage (BAL). The mice were exposed to 14 days of CS or air, and the percentage of CD86- and CD206-positive cells in BAL was determined by flow cytometry. All statistical analyses were performed with a one-way ANOVA, followed by a Tukey’s *post hoc* test. The data represent the means ± SEM of 7–10 mice per group. * *p* < 0.05 compared to control.

The expressions of CD206 and CD86 have been used as M1 and M2 markers, respectively. Thus, we investigated the expression these proteins have in AM of mice exposed to CS. In the control group, there was a predominantly CD86^+^, M1 macrophage population (Figure 5). However, we identified fewer CD86^+^ macrophages and an increased number of CD206^+^ cells after CS exposure for 14 days (Figure 5). These results confirmed that CS can skew M2 macrophage polarization in vivo after 14 days of CS exposure.

### 2.4. Effects of the CS Profile of Monocytes in the Circulation and in the Bone Marrow after Cigarette Smoke Exposure

**Figure 6:**
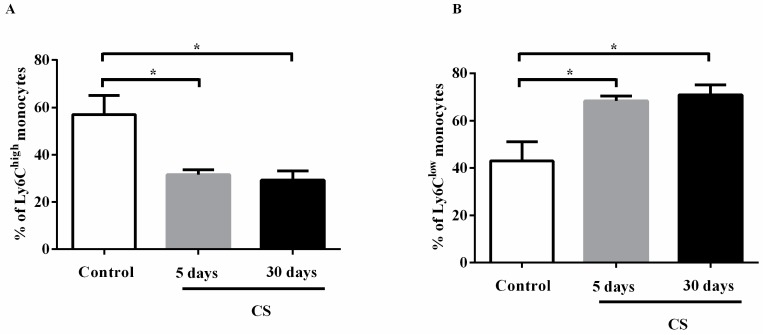

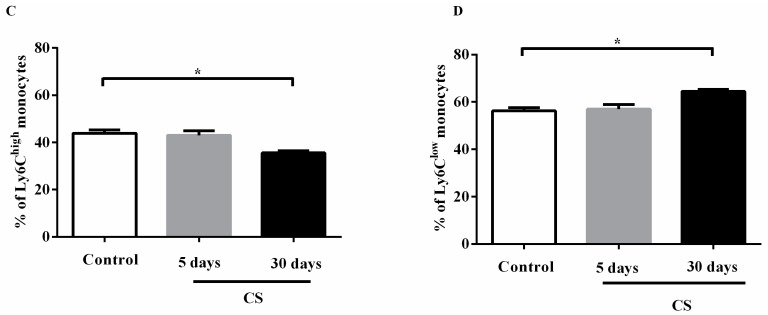
Effects of CS on the % of Ly6C^high^ (**A** and **C**) and Ly6C^low^ (**B** and **D**) monocytes in blood and bone marrow of mice. The percentage of Ly6C^high^ (classical) monocytes and Ly6C^low^ (non-classical) monocytes was determined by flow cytometry. All statistical analyses were performed with a one-way ANOVA, followed by a Tukey’s *post hoc* test. The data represent the means ± SEM of 7–10 mice per group. * *p* < 0.05 compared to control; * *p* < 0.05 compared to 5 days group.

Based on the expression of Ly6C cell surface markers, we investigated the monocyte phenotype in blood and bone marrow of mice exposed to CS. The results revealed that CS exposure decreased the number of Ly6C^high^ cells in blood after 5 and 30 days of exposure (Figure 6A) and consequently increased the number of Ly6C^low^ cells (Figure 6B). Moreover, CS can change the monocyte phenotype in bone marrow, with an increase in Ly6C^low^ cells, but only in the CS 30 days group (Figure 6C,D). These results showed for the first time that CS can change not only macrophage polarization but also monocyte phenotype in blood and bone marrow.

## 3. Discussion

The molecular and cellular mechanisms by which CS causes the inflammatory process and pathogenesis of COPD is still not fully elucidated. In this study, we confirmed that short CS exposure (5 days) induced acute pulmonary inflammation with a pronounced influx of neutrophils and macrophages in the lung associated with increased levels of KC, TNF-α, NO and MMP-12. After 14 days of CS exposure, the neutrophil recruitment and cytokine and chemokine production were greatly reduced. Moreover, CS chronic exposure leads to development of chronic pulmonary inflammation with increased recruitment of macrophages and TGF-β production as well as no detection of TNF-α, IL-6 and KC. These events were associated with the time-course changes in AM, blood monocyte, and bone marrow monocyte phenotypes, inducing a M2 and no classical monocyte phenotype.

The current findings are in agreement with other studies that reported an increase of neutrophils and macrophages in the lung and BAL after short times of exposure to cigarette smoke [27,28]. Some studies show a decrease in these cells after 30 days [29] or 60 days of exposure in mice [30] when compared to a short exposure. We also observed a marked recruitment of inflammatory cells also into the BAL (Supplementary Material Figure S1), consisting of a major number of neutrophils in 5 days when compared to the control group or the CS 30 days group. However, higher numbers of macrophages and lymphocytes were observed in the CS 30 days group when compared to the control or CS 5 days groups, as shown in lung tissue and previously described [29]. This suggests that chronic exposure of the airways to CS results in a mixture of acute and chronic inflammatory response. The current results can be explained by both an innate and an adaptive immune response to CS [3].

Other studies have shown a number of mast cells and macrophages in the epithelium, which was significantly increased in patients with COPD, but neutrophil and T cell numbers did not differ between the groups [4]. In contrast, Hogg et al. reported that progression of COPD was associated with increasing infiltration of the airways by neutrophils, macrophages, CD4 cells, and lymphocyte subtypes [3]. Inflammatory cell recruitment was associated with increased levels of KC, TNF-α and IL-6, which are involved in the chemotaxis and activation of neutrophils and monocytes. Based on this association of macrophages with inflammation in COPD, one would expect that macrophages in COPD are polarized toward the proinflammatory M1 phenotype due to the increase of proinflammatory mediators that are a characteristic of COPD. However, different studies showed that CS decreased the release of cytokines in basal macrophages and/or induced by lipopolysaccharide (LPS) in vitro [18,31], except IL-8. Other studies indicated that macrophages have reduced proinflammatory M1 phenotype following exposure to CS extract in vitro [18,32]. BAL from smokers expressed higher numbers of cells than that of non-smokers, but with lower IL-6 concentrations [33] and a depressed capacity for LPS-induced TNF-α and IL-6 release. In line with this study, Shaykhiev et al. reported that AM gene expression to M1 polarization is decreased in macrophages from smokers, and they revealed an unusual M2 polarization program in these cells obtained from bronchoalveolar lavage [14].

We observed that AM after CS 14 days of exposure specifically expressed higher levels of CD206, which is a characteristic marker of the M2 macrophage phenotype [15,16,17]. These results, in combination with the kinetics of nitrite concentrations (increased at 5 days and decreased at 14 days, with no more nitrite observed after 30 days of CS exposure) indicated that the effect caused by CS in macrophages would weaken the ability of macrophages to kill invaders, which are the most likely initial reasons for M2 polarization. Indeed, we observed an increased CD206 (M2 marker) and decreased CD86 (M1 marker) expression in AM after 14 days of CS exposure.

We suggest that in this in vivo model, in the first stage, CS triggers an intense inflammatory response mediated by alveolar macrophages and epithelial cells, releasing chemokines responsible for neutrophils and more macrophage recruitment as previously shown [29,34]. Moreover, this acute response inflammatory is regulated with time through a modulation directing the macrophages to an immunoregulatory phenotype. Our data suggest that in response to CS exposure, tissue resident macrophages initiate an innate immune response by producing the key cytokines KC and TNF-α, amplifying the inflammatory response. Afterwards, the same stimuli drive the process to remodeling tissue favored by the adaptive immune response with M2 phenotype. CS exposure leads to an increase in anti-inflammatory cytokines rather than in the control group, which would result in a lower number of neutrophils resolution. Perhaps this can explain the minor number of these cells in the 30 days of CS group than in the control group.

Proteases are among the numerous mediators released by inflammatory cells after CS exposure. Upon activation, macrophages are a rich source of matrix metalloproteinases, which can degrade extracellular matrix components [35]. We observed an increase of MMP-12 expression after 5 days of CS exposure as previously shown by Le Quement et al. [34]. Moreover, the increase in MMP-12 is associated with the increase of macrophages and their polarization in a M2 phenotype, potentiating the action of matrix degradation [36]. The involvement of MMPs in CS-induced lung inflammation can be mediated by the release of TNF-α from macrophages with the subsequent neutrophil influx. This may explain the increased MMP-12 after 5 days of CS exposure. Although some authors have designated MMPs as a characteristic of M2 macrophage polarization, currently, it is not clear whether MMPs in the setting of COPD had a distinctive M2 signature, as MMPs show both proinflammatory characteristics, with tissue-destructive potential, and tissue remodeling [14].

CS induces influx of inflammatory cells from the blood circulation into the airways. Among all the inflammatory cells, alveolar macrophages play a pivotal role in the pathogenesis of COPD. Blood monocytes are well-characterized precursors of macrophages. Differential expression of Ly6C in monocytes was used to classify them into two subsets: Ly6C^high^ and Ly6C^low^ cells, which are called classical monocytes and non-classical monocytes, respectively. Both Ly6C^high^ and Ly6C^mid^ monocytes respond to proinflammatory stimuli, and they are recruited to lesion sites [6]. In the absence of inflammation, Ly6C^low^ monocytes enter tissues and renew the tissue resident macrophage and dendritic cell populations [24]. Circulating monocytes also have substantial heterogeneity of phenotypes, probably linked to macrophage phenotypes [6,26]. Modulation of monocyte phenotype could have implications for tissue populations of macrophages, and we therefore showed changes in AM phenotypes after CS exposure; an increase of Ly6C^low^ cells was also time-course dependent. These findings appear to suggest that monocytes from blood and bone marrow may help distinct renewing macrophage population subsets in lungs.

This model of mice has limitations, indeed COPD in humans is caused after 20–40 years of cigarette smoke consumption (in general, 1–2 packs/day), while mice have an average life time of 18 months. Moreover, this cigarette smoke exposure model is very heavy like a human who smokes 3–4 packs of cigarettes/day with main hallmark of COPD present in the mouse lung emphysema, after 60 days of exposure. Moreover, the majority of biological functions modulated by cigarette smoke in humans were also affected in mice [37]. Therefore, this data allows us to conclude that in addition to changes in the profiles of resident pulmonary macrophages by direct exposure to CS, our data suggest that CS also leads to changes in the profile of circulating monocytes before migration and recruitment. These new data on the profile changes of monocytes in the blood can be used as a systemic marker of susceptibility to cigarette-smoke-induced lung injury. Moreover, future studies are needed to clarify whether the Ly6C^low^ monocytes in the blood and bone morrow are the M2 macrophages in the lungs.

## 4. Materials and Methods

### 4.1. Cigarette Smoke Exposure

Adult male C57BL/6J mice (6–7 weeks old) were purchased from the Instituto de Veterinária/Universidade Federal Fluminense (Niterói, Brazil). The mice were maintained under controlled conditions with 12 h light/dark cycle and had free access to food and water during the experiment. All experimental animal work was approved by the Ethical Committee for Animal Use of State University of Rio de Janeiro (CEUA/009, 29 March 2016). To study the effects of CS, mice were exposed to 12 commercial full-flavored Marlboro cigarettes per day for 5, 14 and 30 days with the use of a smoking chamber, as described previously [27,28]. Briefly, animals were placed in the inhalation chamber (40 cm long, 30 cm wide and 25 cm high) inside an exhaustion chapel. A cigarette was coupled to a plastic 60 mL syringe so that puffs could be drawn in and subsequently expelled into the exposure chamber. One liter of smoke from 1 cigarette was aspirated with this syringe (20 puffs of 50 mL), and the puff was immediately injected into the chamber. The mice were maintained in this smoke-air condition (∼3%) for 6 min. Then the cover was removed from the inhalation chamber, and by turning on the exhaustion of the chapel, the smoke evacuated within 1 min. This cigarette exposure was repeated 4 times in succession for 3 times per day (morning, lunch time, and afternoon) resulting in 72 min of cigarette-smoke exposure from 12 cigarettes/day. This protocol was first performed by us and it is efficient to induce lung inflammation Mice (*n* = 47) were assigned to the following five groups: control group (sham mice) 14 days (*n* = 7), control group 30 days (*n* = 10), CS 5 days group (*n* = 10), CS 14 days group (*n* = 10), and CS 30 days group (*n* = 10). The control groups were exposed at the same procedure of CS group. Thus, three times per day the mice of the control group were placed in the inhalation chamber and exposed to room air for 28 min (equals 4 cigarettes 6 min each) with interval of 1 min of exhaust after each exposure air by 14 days or 30 days. No difference was observed in control groups after 14 days and 30 days of the air exposure. The groups CS 5 days and CS 14 days were compared to control group 14 days of air exposure and CS 30 days were compared with control of 30 days of air exposure.

### 4.2. Analysis of Bronchoalveolar Lavage Fluid

One day after the last exposure to CS, the mice were sacrificed by cervical dislocation. After incision of the trachea, a plastic cannula was inserted, and airspaces were washed using 2 mL of phosphate buffer solution (PBS) (4 times with 0.5 mL). The rib cage was then gently massaged to enable maximum cell collection. The BAL fluid was collected by careful aspiration and then centrifuged (600× *g* for 10 min), and supernatant was collected and stored at −80 °C. The cell pellet from mice exposed to CS for 5 and 30 days was then re-suspended in 0.4 mL of PBS for cell counting, and the total number of cells was determined using a Neubauer chamber.

### 4.3. Phenotype Analysis by Fluorescence-Activated Cell Sorting (FACS)

After the BAL procedure, the chest was opened and the blood sample was collected from the heart by cardiac puncture. The bone marrow-derived monocytes were flushed from the femurs with PBS by inserting a 27-gauge needle in femurs attached to a 10 mL syringe containing 1 mL of PBS. Then, 200 µL samples were incubated with 2 mL of red cell lysis buffer (BD Biosciences, San Diego, CA, USA) for 10 min, and the tubes were centrifuged at 500× *g* for 5 min. The supernatant was discarded, and the pellet was washed in PBS, centrifuged again for 5 min, re-suspended in 500 μL of 70% ethanol and stored at −2 °C. Aliquots of 100 μL of cells suspended in PBS were stained with the indicated antibodies prior to fluorescence-activated cell sorting (FACS) analysis.

The reprogramming of AM polarization correlates with CS in patients with COPD [14,15]. We examined whether and when CS influences the macrophage and monocyte phenotype in mice. Flow cytometry was used to detect the lung alveolar macrophage phenotype. Single-cell suspensions were analyzed by 3-color flow cytometry. Cells from BAL were incubated with fluorescein isothiocyanate-conjugated (FITC) anti-CD206 (clone C068C2 from Biolegend, San Diego, CA, USA), phycoerythrin-conjugated anti-f4/80 (clone BM8 from Biolegend, San Diego, CA, USA) and APC-conjugated anti-CD86 (clone PO3 from Biolegend, San Diego, CA, USA). Cells from blood and bone marrow-derived monocytes were incubated with PE-anti Ly6C (HK1.4 from Biolegend, San Diego, CA, USA) or isotypic controls for 20 minutes at 4 °C and analyzed with FACSCalibur cytometer (BD Biosciences, San Diego, CA, USA). Flow cytometry data were analyzed using Cellquest Pro software (BD Biosciences, San Diego, CA, USA). Monocyte cells were first gated according to their forward and side-scatter profiles, and then macrophages were defined as CD206^+^ or CD86^+^ and monocytes as Ly6C^high^ or Ly6C^low^. First, the % of cell markers was calculated in the control group and afterwards was calculated in CS group.

### 4.4. Tissue Processing

After blood collection, the right ventricle of each mouse was perfused with saline and the right lung was harvested and fixed buffered formalin (10%, pH 7.2) for 24 h for histology analysis. The left lung was homogenized in RIPA buffer (20 mM Tris/HCl, 138 mM NaCl, 10% glycerol and 1% Triton) containing 1% protease inhibitor cocktail and phosphatase inhibitor cocktail (Roche, Mannheim, Germany) and centrifuged. The supernatant was stored at −80 °C. The total protein in the samples (tissues and BAL) was determined with bicinchoninic acid (BCA) colorimetric assay (Thermo Scientific, Rockford, IL, USA). The lung proteins were analyzed for quantification MMP-12 (H-52; Santa Cruz Biotechnologies, Santa Cruz, CA, USA) and total β-actin (CP01; Calbiochem, San Diego, CA, USA) by Western blotting.

### 4.5. Histology and Immunohistochemistry

The lung lobe was sectioned into 3 portions (3–4 mm) and embedded in paraffin. Sections were stained with hematoxylin–eosin (Sigma-Aldrich, St Louis, MO, USA) using the standard protocol. To quantify neutrophils and macrophages, lung sections were respectively immunolabeled with rat monoclonal anti-myeloperoxidase (#71674; Santa Cruz Biotechnology, Santa Cruz, CA, USA; 1:400) and rat monoclonal anti-F4/80 (Serotec Inc., Raleigh, NC, USA; 1:400) antibodies. We followed the protocol of Romana-Souza et al. [38] briefly, for antigen retrieval, sections were incubated with citrate buffer (pH 6.0) before labeling then endogenous peroxidase was inhibited, sections were incubated for 45 min at 24 °C in a moisty chamber primary antibody and after washing, revelation was performed using anti-rat secondary antibody followed by incubation with streptavidin (DAKO, Carpinteria, CA, USA) and diaminobenzidine was used as the chromogen. Sections were counterstained with hematoxylin. No labeling was observed on sections where the primary antibody was omitted. To quantify the number of immunostained cells, ten random fields per animal (14,689 μm^2^) were analyzed as described by Romana-Souza et al. [38].

### 4.6. Analysis of Cytokines, Chemokines and Nitric Oxide

TNF-α (#555268; BD Biosciences, San Jose, CA, USA), active TGF-β1 (#559119; BD Biosciences, San Jose, CA, USA), (KC) (#558266; BD Biosciences, San Jose, CA, USA) and IL-6 (#559331; BD Biosciences, San Jose, CA, USA) were measured in lung lysate using a sandwich ELISA kit. All procedures were performed according to the manufacturer’s instructions. The results are expressed as pg per mg of total protein. To evaluate nitric oxide (NO) synthesis, nitrite levels were measured in lungs lysate samples after incubation with Griess reagent as described [30].

### 4.7. Western Blotting

Total protein (30 µg/lane) from lung homogenate was subjected to 8 or 10% SDS-PAGE and transferred to nitrocellulose membranes. Membranes were then washed once with Tris/HCl, pH 7.4, containing 159 mM NaCl and 1% Tween 20, Tris buffered saline (TBS), and then blocked with 5% non-fat milk (Nestlé, São Paulo, Brazil). After, the membranes were washed with TBS and probed with MMP-12 (H-52; Santa Cruz Biotechnologies, Santa Cruz, CA, USA). β-actin (#5441; Sigma-Aldrich, St. Louis, MO, USA; 1:1000) was used as a loading control. After washing with TBS-T, following incubation with the appropriate horseradish peroxidase-conjugated secondary antibodies (1:2000), after washing with TBS, immunoreactive protein bands were revealed using an enhanced chemiluminescence assay (Amersham Pharmacia, Little Chalfont, UK). The results are expressed as arbitrary units.

### 4.8. Statistical Analysis

The results are expressed as the means ± SEM. Analysis of CS effects between groups was performed with one-way ANOVA. All analyses were performed using Prism5 (GraphPad Software; San Diego, CA, USA). Comparisons were made between the control and CS groups; the groups CS 5 days and CS 14 days were compared to control group 14 days of air exposure and CS 30 days were compared with control of 30 days of air exposure. Statistical testing was performed using Tukey’s *post hoc* test. For each analysis, *p* values less than 0.05 were considered statistically significant.

## 5. Conclusions

COPD is a highly heterogeneous disease, and we propose that two different macrophage phenotypes predominate at different times. We observed the M1 phenotype in the early stages of CS exposure and the M2 phenotype at later stages in COPD. Finally, macrophage reprogramming can be a perspective therapeutic strategy for COPD.

## Supplementary Materials

Supplementary materials can be found at http://www.mdpi.com/1422-0067/18/9/1940/s1.

## Abbreviations

AM	alveolar macrophages
BAL	bronchoalveolar lavage
BCA	bicinchoninic acid
COPD	chronic obstructive pulmonary disease
CS	cigarette smoke
IL	interleucin
KC	keratinocyte chemoattractant
MMP	matrix metalloproteinase
MPO	myeloperoxidase
NO	nitric oxide
ROS	reactive oxygen species
TGF-β	transforming growth factor-β
TNF-α	tumor necrosis factor-α
