# Supplementary material for: Time Course of the Phenotype of Blood and Bone Marrow Monocytes and Macrophages in the Lung after Cigarette Smoke Exposure In Vivo
Source: Int J Mol Sci. 2017 Sep 9;18(9):1940. doi: 10.3390/ijms18091940 (PMC5618589; doi:10.3390/ijms18091940)
Supplement: Supplementary file 1 [file ijms-18-01940-s001.pdf]

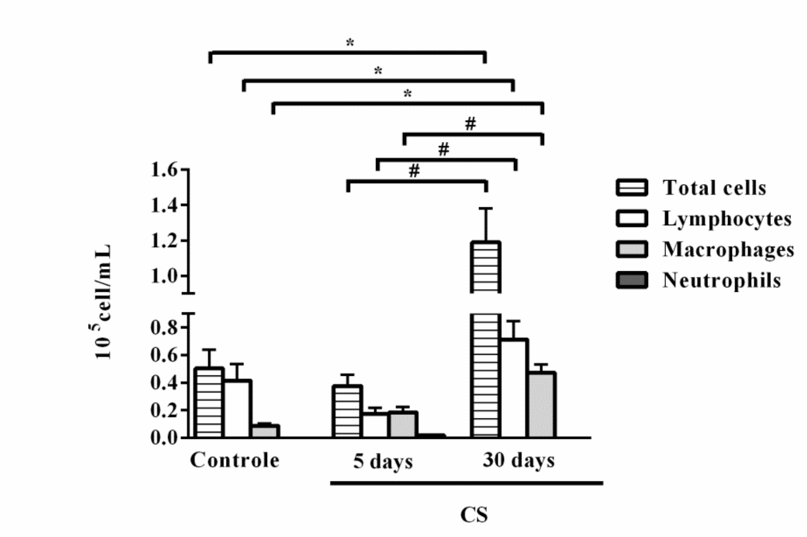

Supplementary Figure S1. Effects of cigarette smoke exposure on total and differential numbers of cells in BAL. The mice were exposed for 5 days or 30 days to CS or air, and BAL was isolated. All cell types were enumerated by cytospin counts. * $p < 0.05$ when compared to control group; # $p < 0.05$ when compared to CS 5 days group. Data are presented as the means ± SEM of 7–10 mice per group. All statistical analyses were performed with a one-way ANOVA, followed by a Tukey's *post hoc* test.
